# Clinicopathologic characteristics and treatment outcomes of pancreatic cancer patients at a tertiary referral hospital in Kenya

**DOI:** 10.3332/ecancer.2024.1682

**Published:** 2024-03-19

**Authors:** Sheila W Muchiri, Elly O Ogutu, Peter O Oyiro, Lars Aabakken

**Affiliations:** 1 Department of Clinical Medicine and Therapeutics, University of Nairobi, Nairobi 00100, Kenya; 2 World Gastroenterology Organisation Nairobi Training Center, Nairobi 00100, Kenya; 3 Institute of Clinical Medicine, Oslo University Hospital-Rikshospitalet, Oslo 0318, Norway

**Keywords:** pancreatic neoplasms, retrospective studies, treatment outcome, survival rate, Kenya

## Abstract

The global incidence of pancreatic cancer (PC) continues to steadily increase whereas its prognosis remains poor. Previous studies have suggested worse outcomes among individuals of African descent. The characteristics of patients with PC in Kenya, and their contemporary management and survival outcomes remain largely unknown. This study aimed to describe the clinical and pathologic characteristics, management, and outcomes of patients diagnosed with PC at Kenyatta National Hospital (KNH), a tertiary referral hospital in Kenya. Records of 242 patients diagnosed with PC at KNH between 1st January 2014 and 30th September 2021 were assessed in this retrospective cohort study. Data on their clinical, histopathologic, and treatment characteristics was presented as mean (± standard deviation) and/or median (interquartile range) for continuous variables and frequency (percentage) for categorical variables. Kaplan–Meier and Cox proportional hazard ratios were used for survival analysis. PC occurred in a young population, the median age being 58.5 years (inter-quartile range 35–88). The majority of tumours (54%) were metastatic at diagnosis, while 28% and 14% were stage III and stage I/II, respectively. Surgical resections with curative intent were performed on 7% overall and 44% of stage I/II cases. The majority of patients with stage I/II disease (52.9%) received chemotherapy whereas the majority with stage III and IV disease received the best supportive care only (62.7% and 64.9%, respectively). Patients who underwent surgical resection (HR for mortality 0.20, 95% CI 0.05–0.83, p = 0.021) and chemotherapy (HR for mortality 0.15, 95% CI 0.08–0.29, p < 0.001) had significantly improved survival, reflecting a more favourable stage of the disease more amenable to aggressive therapies. The median survival time was 3 months and the 1-year survival rate was 32%.

## Background

Despite its relatively low incidence, pancreatic cancer (PC) is among the leading causes of cancer mortality worldwide [1]. It is currently the seventh most common cause of cancer deaths and is set to be the second leading cause by 2030, due in part to the improvement in survival rates observed in other more common cancers [2, 3]. Notably, the greatest increase in incidence globally is projected to occur in Africa [4]. Although current literature suggests that the incidence and mortality rates of PC in Eastern Africa are some of the lowest in the world, the data from this region likely underestimates the burden of disease, reflecting limitations in diagnostic capacity for parts of this population, and the incompleteness of national cancer registries [4]. There is evidence that individuals of African descent not only have a considerably higher risk of PC than any other racial group but also have a younger age of disease onset, more advanced stage at diagnosis and a higher mortality rate [5, 6]. The reasons for this racial disparity are not completely understood.

PC often presents at a late stage, contributing to a dismal 5-year survival rate ranging from as low as 2% in some countries to 9% [4, 7]. Further, limitations in access to care with delayed referrals to oncology and lower rates of surgical resection have been linked to the higher mortality rates observed in low-resource populations including blacks [5]. Whereas historically there was little variability in survival outcomes, there is a widening gap in survival between patients with resectable PC and those with unresectable disease, on the back of several therapeutic advancements made in the last three decades [2, 8, 9]. Advances in surgical techniques have resulted in dramatically improved surgical outcomes as well as expansion in the proportion of tumours amenable to resection due to increasing capabilities in vascular reconstruction [9, 10]. Additionally, the use of multiagent neoadjuvant and adjuvant chemotherapy has been demonstrated to improve survival of patients with borderline resectable and locally advanced tumours [8, 11–14]. There is limited data on the uptake of these treatment modalities in Sub-Saharan Africa. Earlier Kenyan studies indicated a predominatly palliative approach to the management of PC [15, 16]. In view of the rising burden of PC, there is a need for up-to-date local data on the epidemiologic and mortality trends in Kenya. This study aimed to characterise the clinical and tumour features as well as the treatment and outcomes of patients diagnosed with PC in a Kenyan population.

## Methods

### Study design

This was a retrospective cohort study using data from medical records of the Kenyatta National Hospital (KNH) between January 2014 and September 2021. Records of patients with a diagnosis of PC (International Classification of Diseases for Oncology-10th edition code C25) made between 1 January 2014 and 30 September 2021 were reviewed. The case definition of PC was based on a diagnosis made using computed tomography and/or magnetic resonance imaging, positron emission tomography, or endoscopic ultrasonography, with or without cytological or histological confirmation of ductal adenocarcinoma of the pancreas. Those with benign and premalignant, neuroendocrine, acinar cell and periampullary tumours, and non-pancreatic tumours metastatic to the pancreas were excluded from the analysis. Cases without a record of cross-sectional imaging to make the diagnosis of PC were also excluded from the study. Microscopic diagnosis was not required since there is consensus that in the presence of a solid pancreatic mass suspicious for malignancy, biopsy proof is not required before resection [13, 17]. Data on age, sex, risk factors including alcohol and cigarette use, family history of cancer, comorbidities, presenting features, tumour characteristics, cancer staging and treatment were collected. Based on the radiological and clinical information documented in the files, patients were categorised into three stages, defined according to the American Joint Committee on Cancer/Union for International Cancer Control (AJCC/UICC) TNM staging system, 8th edition (Appendix A) [18]. Eastern Cooperative Oncology Group (ECOG) performance score was categorised into the following groups ‘0–1’, ‘2’ and ‘3+’. Surgical resection was defined as excision of the primary tumour with curative intent. Post-operative mortality was calculated at 30 and 90 days following surgery. The assignment of oncologic treatment categories as neoadjuvant, adjuvant or palliative was based on clinicians’ notes and the timelines with respect to surgery. Chemotherapy and/or radiotherapy were considered palliative if no resection was done. Patients who received neither surgical resection nor chemotherapy were classified as having received best supportive care only. Survival time was defined as the time from the date of diagnosis to the date of death or last follow up. Patients’ vital status was recorded as documented in their files on the date of last contact. In cases where the vital status at 1-year post-diagnosis was not known, they were recorded as ‘lost to follow-up’. The data collection tool is illustrated in Appendix B.

### Data analysis

The primary objective was to describe the clinical, tumour and treatment characteristics and the survival outcomes of patients with PC. Data were presented as mean (± standard deviation) and/or median (interquartile range) for continuous variables and frequency (percentage) for categorical variables. Patient demographics, tumour characteristics and treatment modalities were compared among the stage categories using Fisher’s Exact or Pearson chi-square test for categorical variables and analysis of variance for continuous variables. The number of pancreaticoduodenectomies performed in each year was used to produce a line graph illustrating the temporal trend. The Kaplan–Meier method was used to analyse survival probabilities by treatment modality, and the differences were compared using the log-rank test. The study was approved by the institutional review board of the KNH/University of Nairobi. Statistical analysis was performed using STATA. Statistical significance was defined by two-sided *p* < 0.05.

## Results

### Clinicopathologic characteristics

A total of 242 patients were enrolled, the majority of whom 129 (53%) were female and 113 (47%) were male. The median age was 58.5 years (IQR 35–88; range 32–92).

The most common presenting features were abdominal pain, present in 73%, jaundice in 68%, features of cholestasis-pruritus, pale stools, dark urine in 38% and weight loss in 30% of cases. Other clinical features at presentation were loss of appetite (26.5%), and back pain (8.7%). The median duration of symptoms at presentation was 3 months (IQR 1–18; range 0.5–24 months).

Family history of cancer was present in 14 (5.8%) of patients, none of whom had a documented family history of PC. History of alcohol use was positive in 58 (24%) of cases whereas 57 (24%) had a documented history of smoking. History of chronic pancreatitis was present in four patients (1.7%). Forty-five patients (18.6%) were known to have diabetes mellitus (DM), and of the 38 patients with documented duration since diagnosis of diabetes, 24 (36%) were diagnosed within 12 months of diagnosis of PC.

Thirty-four (14%) patients had stage I/II disease, 67 (28%) had stage III disease and 131 (54%), had stage IV disease at diagnosis. There was no staging information for 10 (4%) patients. For patients with stage III/unresectable disease, the reason for unresectability was arterial and/or venous invasion in 32 (48%) of cases, extra-pancreatic local extension in 21 (31%) and regional lymph node extension in 9 (13%).

The majority of patients, 196 (81%) had tumour involving the head of pancreas, whereas 46 (19%) had tumour involving the body and/or tail of the pancreas. Tumours involving the pancreatic head were dominant in stage I/II disease (97.1%) compared to stage III disease (80.6%) and stage IV disease (76.3%) whereas the occurrence of tumours in the body or tail of the pancreas was higher in advanced disease; 2.9% of stage I/II versus 19.4% of stage III and 23.7% of stage IV (All* p* = 0.013) (Table 1).

A total of 94 (38.8%) had their celiac axis (CA) 19–9 documented at diagnosis. Pre-treatment CA 19–9 levels were normal (<37 U/mL) in 26 (10.7%) cases out of the entire population and in 14 (11.4%) cases with histologically confirmed pancreatic ductal adenocarcinoma (PDAC).

Of those who had their performance status (PS) documented, the majority had relatively good performance scores: ECOG 0–1 in 73 (46.2%), ECOG 2 in 37 (23.4%) whereas 48 (30.4%) had poor performance scores of ECOG 3–4.

Of the 242 patients, 146 (60%) had a histologic or cytologic diagnosis, of which 123 (84%) confirmed PDAC. Five (29%) of the 17 patients who underwent pancreatoduodenectomies had pre-operative histologic confirmation of PDAC. Of the patients with histologically confirmed PDAC, 16 (13%) had well differentiated tumours whereas 62 (50%) had moderately or poorly differentiated tumours.

### Treatment

A total of 17 patients (7%) underwent radical resection. These included 15 of the 34 (44%) patients who presented initially with stage I–II tumours and 2 of the 67 (3%) patients with stage III tumours at diagnosis, who underwent resection after having been down-staged to resectable disease with neoadjuvant chemotherapy. All resected tumours involved the head of the pancreas and all resections were pancreaticoduodenectomies, with no distal or total pancreatectomies. There was no documentation of vascular resection. Of the remaining patients initially diagnosed with stage I/II disease, nine were found to have unresectable disease intraoperatively, whereas five declined surgery and five were lost to follow-up after their initial diagnosis. A total of 70 patients underwent surgery, of whom 40, representing the majority at 57% had palliative procedures whereas 13 patients (19%) had exploratory surgery only. Among the 17 patients who underwent radical surgery, there was 1 mortality (5.8%) in the 30-day post-operative period and none recorded in the 90-day post-operative period.

**Figure 1.**
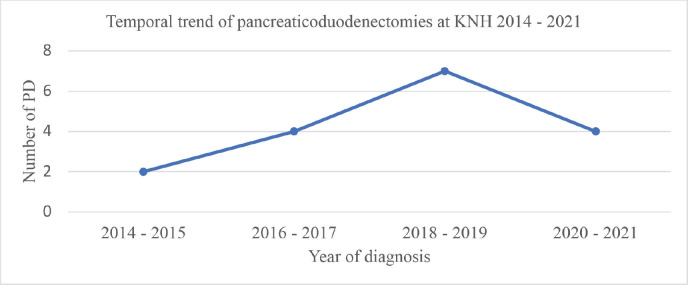
Temporal trend of pancreaticoduodenectomies at KNH 2014–2021. PD, pancreatoduodenectomies.

Analysis of the temporal trend in surgical resection over the 8-year period of this study revealed an increase in the number of pancreatoduodenectomies conducted in 2014–2015, 2016–2017 and 2018–2019 with 2,4 and 7 cases for each of these respective periods. There was subsequently a decrease during the 2020–2021 interval to 4 cases. This trend is illustrated in Figure 1.

Of the 242 patients, 88 (37%) received at least one cycle of chemotherapy. Assessed by stage, 18 (52.9%) patients with stage I/II disease received chemotherapy, as did 24 (35.8%) of patients with stage III disease and 46 (35.1%) of patients with metastatic disease (Table 1).

Among the 101 patients with non-metastatic disease, a total of 12 (12%) patients were offered neoadjuvant chemotherapy with the intention to subsequently undergo surgery. The most common regimen was FOLFIRINOX, given in 6 (50%) of cases, followed by gemcitabine plus oxaliplatin in 3 (25%), gemcitabine in 2 (17%) and FOLFOX in 1 (8%) case. The median number of cycles was 5 (IQR 3–6). Of these patients, nine had stage I/II disease whereas three had stage III disease at diagnosis. Subsequently, only five of these patients went on to have surgery; two patients underwent pancreatoduodenectomy whereas three were found to be inoperable intra-operatively. The remaining seven did not undergo surgery, either because they declined operative management or because they were lost to follow-up. Pre-operative chemotherapy was therefore in effect administered to 5 (4.9%) of patients with non-metastatic disease.

Nine patients (53%) out of 17 who had surgical resection received adjuvant chemotherapy, for a median of 6 cycles, (IQR 4–8). Of these, seven had stage I/II disease whereas two had stage III disease at diagnosis. One had also received pre-operative chemotherapy while the rest received adjuvant chemotherapy alone. Four patients (44%) received adjuvant FOLFIRINOX whereas 3 (33%) received gemcitabine plus cisplatin and 2 (22%) gemcitabine plus oxaliplatin. No patients received adjuvant chemoradiation.

Palliative chemotherapy was administered to 70 (29%) patients, for a median of 3 cycles, (IQR 1–11; range 1–20). The most common chemotherapy regimen, administered in 47 (40.5%) cases was FOLFIRINOX. Gemcitabine in combination with capecitabine, cisplatin or oxaliplatin was given in 42 (36%) cases whereas in 6 (5%) cases gemcitabine was given alone. In 18 (16%) cases patients received capecitabine either alone or in combination with oxaliplatin. FOLFOX was administered in 3 (2.5%) cases. One patient with stage III disease received concurrent chemoradiation with capecitabine.

Overall, 154 (63.6%) patients did not receive chemotherapy. For 27.6% of these patients, this was due to loss of follow-up, with no repeat visit following their first admission to KNH. Twenty percent of patients with multiple repeat visits had no documented reason for non-administration of chemotherapy. Among patients with documented reasons for not offering chemotherapy, in 24% of cases, it was related to early death; in 23% it was due to poor PS and/or old age. In 4% of cases, patients refused chemotherapy whereas 1.3% had a contraindication.

Overall, 146 (60%) patients received best supportive care only. There was a significant difference among the three clinical stages, with higher rates among those with advanced disease. Nine (26.5%) of patients with stage I/II disease, 42 (62.7%) of those with stage III and 85 (64.9%) of patients with stage IV disease at diagnosis received best supportive care only (*p* ≤ 0.001). For the palliation of obstructive jaundice, 71 (29.3%) patients underwent endoscopic biliary stenting, whereas 42 (17.3%) and 28 (11.6%) underwent open biliary-enteric bypass and percutaneous transhepatic cholangiography respectively. The majority of patients with gastric outlet obstruction underwent open gastrojejunostomy, performed on 28 (11.5%) patients whereas 1 (0.4%) had an enteral stent placed endoscopically. Three (1.2%) patients with metastatic disease received palliative radiotherapy. Other supportive measures included analgesia including opioids, given to 84 (34.7%) and nutritional support, given in 45 (18.6%) cases. Of note, only 28 (11.5%) patients had a documented formal palliative care team review.

### Survival

**Figure 2.**
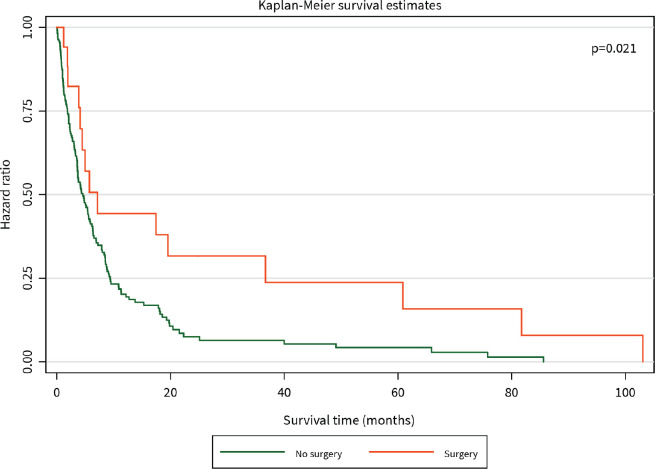
Kaplan–Meier curve of overall survival (months) among PC patients diagnosed between 2014 and 2021 stratified by surgery.

**Figure 3.**
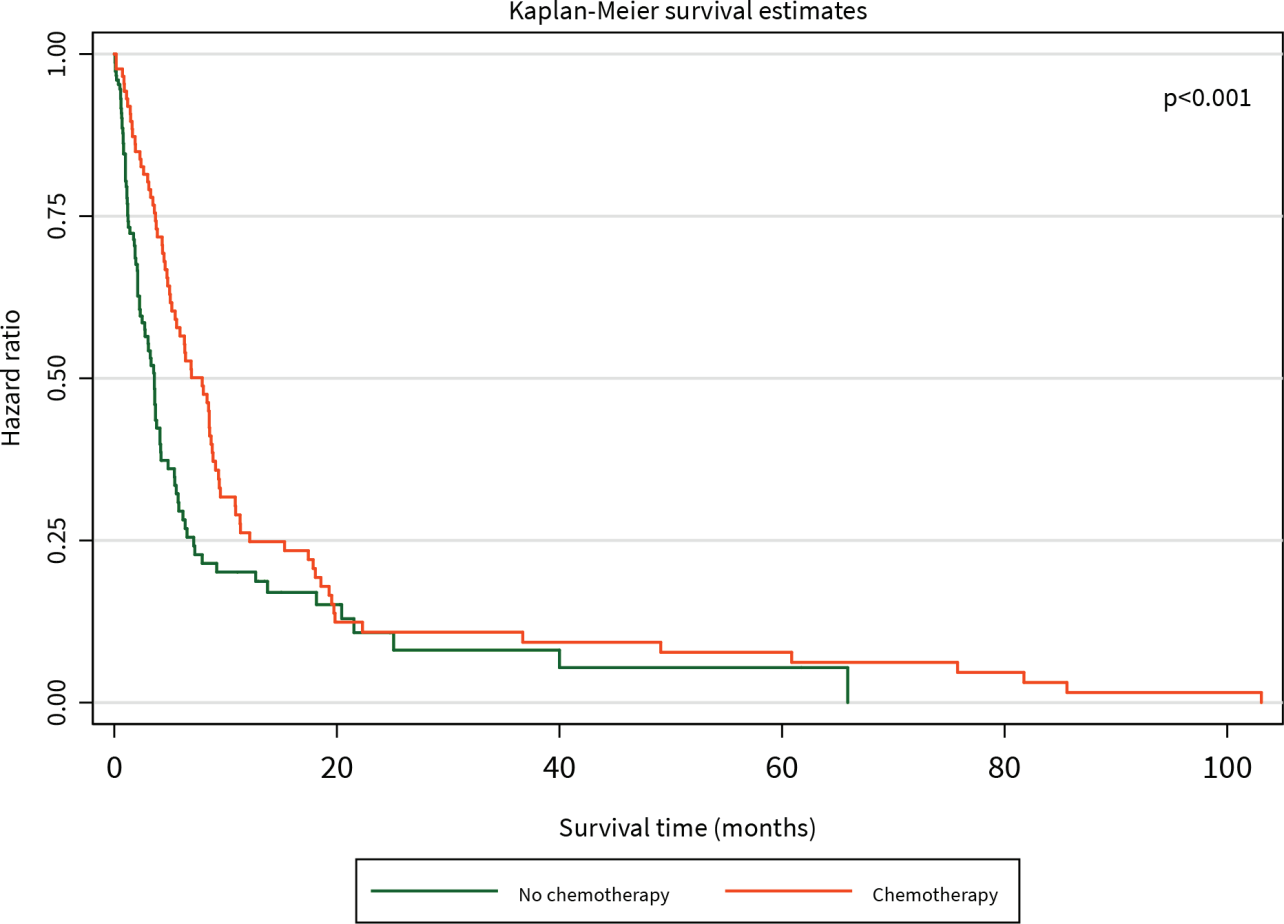
Kaplan–Meier curve of overall survival (months) of PC patients diagnosed between 2014 and 2021 stratified by chemotherapy.

The median survival time was 3 months. The 1-year survival rate was 32%. Patients who had surgical resection with curative intent had significantly better survival than those who did not (HR for mortality 0.20, 95% CI 0.05–0.83, *p* = 0.021), and this benefit persisted for the duration of 105 months until close of the study (Figure 2). Patients who received chemotherapy were also found to have significantly improved survival compared to those who did not (HR for mortality 0.15, 95% CI 0.08–0.29, *p* < 0.001), although this difference was diminished after about 20 months (Figure 3).

## Discussion

This retrospective study reviewed the clinical and pathologic characteristics, management, and outcomes of patients with PC in the setting of a national referral hospital with specialist oncology services in Kenya. The results indicate that PC occurred at a relatively young age in this cohort of patients, with a median age of 58.5 years and the majority (64%) being younger than 65 years. This is similar to the findings of other studies conducted in African populations including Zambia (mean age 55.7 years) [19] and Malawi (mean age 52.1 years) [20] and in African American populations, where it has been found that the age of onset is significantly younger compared to Caucasian patients (63.3 versus 67.1 years) [6]. Similar to other studies, the majority of patients (54%) had metastatic disease at diagnosis. The difficulty in making an early diagnosis of PC is attributable to the disease being initially clinically silent and the presentation with non-specific signs and symptoms [7]. In Kenya, this is compounded by the high cost of diagnostic testing and limited access to cancer services particularly in remote rural parts of the country, both which contribute to poor health-seeking behaviour [21].

The prevalence of alcohol use in the current population (24%) was higher than that of the general Kenyan population, reported as 19%, as was that of cigarette smoking, at 24% compared to the national average of 13% [22]. DM was more prevalent, at 18%, compared to 4% in the general Kenyan population [23]. The higher prevalence of cigarette smoking, alcohol use, diabetes and obesity has been suggested as a possible contributor to the disproportionately higher incidence of PC among African Americans compared to Caucasians as well as its occurrence at a younger age [6]. It is however understood that there are likely multiple reasons for the racial differences in PC incidence and mortality including socio-economic discrepancies limiting access to care; further, the possibility of an underlying genetic aetiology is being explored [5, 6].

There was an increase in the rate of surgical resection compared to historical data from the same institution, at 44% for stage I/II disease compared to none documented in the period spanning 1977–1985 [15] and 1 (2.4%) between 2003 and 2004 [16]. During the 8-year period of this study, there was an initial increase in the number of pancreatoduodenectomies. This is likely multifactorial, resulting from an increase in the number of surgeons with technical capabilities in hepato-pancreato-biliary surgery as well as enhanced capacity of radiology, anaesthesia/critical care, pathology, and oncology, all collaborating in the diagnosis, management of post-operative morbidity and improvement of surgical outcomes. This was, however, followed by a decrease in 2020–2021 which may be explained by the limitations in healthcare access and the constraints on non-emergent operations occasioned by the outbreak of the COVID-19 pandemic as has been widely reported [24, 25]. Low rates of overall resection have been reported in other African studies including a single-centre study in Nigeria which reported a rate of 3% [26], and in Malawi, where a similar study found that 94% of pancreatic resections were palliative [20]. An international study of population-based cancer registries in Europe and the USA reported variable resection rates across countries, ranging from 13.2% to 21.2% overall and 34.8% to 68.7% for stage I–II tumours [27]. In advanced, high-volume centres, the application of vascular resection and the administration of neoadjuvant chemotherapy have been used to achieve resectability rates of up to 60% of previously unresectable tumours [28, 29].

About half the patients with resected tumours were not offered adjuvant chemotherapy. While it is possible that some of these patients may have had poor functional status post-operatively that precluded chemotherapy, in the majority of cases, there was no documented reason for failing to offer chemotherapy and no documented review by an oncologist. Comparable rates of adjuvant chemotherapy have been reported in European countries, where they range from 31% to 52% [30]. An American study based on a national cancer database reported a rate of initiation and completion of adjuvant chemotherapy of 35% and 7%, respectively [31]. Treatment at a high-volume cancer centre has been associated with more guideline-compliant care including the utilisation of multimodal therapy [32]. We noted a gap in interdisciplinary collaboration, with few records of cases having been discussed in a multidisciplinary forum, despite the existing framework to conduct such meetings on a regular basis. This likely accounts for the proportion of cases without the involvement of oncologists.

Both surgery and chemotherapy significantly improved survival, reflecting earlier stage of disease amenable to aggressive treatment. The 1-year survival rate was 32%. In comparison, the global 1-year survival rate of PC is 24% [4]. Few studies on survival outcomes have been conducted in Africa. A retrospective study conducted in Algeria that evaluated 160 patients diagnosed with PC between 2006 and 2013 reported a 1-year survival rate of 20% [33]. Similarly, in a single-centre retrospective study conducted in Nigeria between 1999 and 2013, the 1-year survival rate was 20% [34]. This being a single-centre retrospective study, it may be difficult to compare it to the findings of population-based studies that are able to assess survival prospectively. Moreover, is difficult to exclude the possibility of a confounding effect from patients who received intensive therapy at this referral facility.

A number of limitations are acknowledged. The findings of this study are not generalisable to the entire Kenyan population given that it is based in a national referral hospital providing specialist cancer services. As such, the management and outcomes here cannot be replicated in lower-level facilities. Secondly, the high rate of loss to follow-up limits our ability to conduct a comprehensive survival analysis, given that only about half the population was on follow-up 1 year after their diagnosis. The maintenance of a comprehensive, updated national cancer registry would enhance the collection of quality data and the completeness of follow-up.

## Conclusion

In conclusion, the results of this study indicate that the majority of patients presenting to this tertiary referral facility had advanced disease, limiting the therapeutic options that could be availed to them. This highlights a gap in local capacity at the county level preventing early diagnosis and prompt referral of patients with PC for specialised care. Surgical resection and systemic chemotherapy conferred significant survival benefits. The continued development of Kenyan surgical expertise to expand resectability criteria for appropriately selected individuals would see this benefit afforded to a greater proportion of patients. The management of patients with PC should be embedded in a multidisciplinary approach to allow the accrual of maximal survival benefits from the application of multimodality therapy.

## List of abbreviations

CA, Celiac axis; DM, Diabetes mellitus; ECOG, Eastern Cooperative Oncology Group; KNH, Kenyatta National Hospital; MDCT, Multidetector-row computed tomography; PC, Pancreatic cancer; PDAC, Pancreatic ductal adenocarcinoma; PS, Performance status.

## Conflicts of interest

The authors declare that they have no conflict of interest.

## Funding

No funding was received to assist with the preparation of this manuscript. No funding was received for conducting this study.

## Figures and Tables

**Table 1.** Clinicopathologic characteristics by stage of patients with PC at KNH 2014-2021.

Characteristics	Stage I/II	Stage III	Stage IV	*p*-value
	***n* (%)**	***n* (%)**	***n* (%)**	
Age, mean (±SD), years	55.8 (11.7)	61.7 (11.2)	58.7 (12.3)	0.0509*
Gender				
Male	17 (50.0)	30 (44.8)	60 (45.8)	0.879^†^
Female	17 (50.0)	37 (55.2)	71 (54.2)	
Location				
Head	33 (97.1)	54 (80.6)	100 (76.3)	0.013^∞^
Body and tail	1 (2.9)	13 (19.4)	31 (23.7)	
Treatment				
Surgical resection	15 (44.1)	2 (3.0)	0 (0.0)	0.000^∞^
Chemotherapy	18 (52.9)	24 (35.8)	46 (35.1)	0.162^∞^
Neoadjuvant	9 (26.5)	3 (4.5)	0 (0.0)	
Adjuvant	7 (20.6)	2 (2.9)	0 (0.0)	
Best supportive care only	9 (26.5)	42 (62.7)	85 (64.9)	0.000^∞^

^*^Anova; ^†^Pearson chi-square; ^∞^Fisher’s exact

## Appendix

### Appendix

**Appendix A.** Exocrine PC TNM staging AJCC UICC 8th edition.

**Primary tumour (T)**	
**T category**	T criteria
T1	Tumour ≤2 cm in greatest dimension
T2	Tumour >2 and ≤4 cm in greatest dimension
T3	Tumour >4 cm in greatest dimension
T4	Tumour involves the celiac axis, superior mesenteric artery, and/or common hepatic artery, irrespective of size
Regional lymph nodes (N)	
N category	N criteria
N0	No regional lymph node metastasis
N1	Metastasis in 1–3 regional lymph nodes
N2	Metastasis in ≥4 regional lymph nodes
Distant metastasis (M)	
**M category**	M criteria
M0	No distant metastasis
M1	Distant metastasis
Stage	
IA	T1 N0 M0
IB	T2 N0 M0
IIA	T3 N0 M0
IIB	T1-T3 N1 M0
III	Any T N2 M0 T4 Any N M0
IV	Any T Any N M1

### Appendix

**Appendix B.** Data collection tool- clinicopathologic characteristics and survival outcomes of patients with PC at KNH 2014–2021.

**Sociodemographic characteristics**	
Patient number	
Age	
Sex	
Clinicopathologic characteristics	
Alcohol	
Smoking	
Weight	
Height	
Family history of cancer	
History of chronic pancreatitis	
Co-morbidity	
Diabetes duration	
Symptom duration	
CA 19–9 at diagnosis	
ECOG score	
Imaging modality	
Date of diagnosis	
Tumour location	
TNM stage	
Resectability status	
Tumour size	
N stage	
Metastasis site	
Biopsy method	
Histology	
Grade	
Treatment	
Neoadjuvant chemotherapy regimen/cycles	
Surgery date	
Surgery type	
Adjuvant chemotherapy regimen/cycles	
Palliative chemotherapy regimen/cycles	
Best supportive care interventions	
Radiotherapy	
Treatment response	
Resection margin	
Radiologic restaging	
CA 19–9 post Rx	
Operative complications	
Recurrence date and pattern	
Survival data	
Date of last contact	
Vital status at last contact	
Date of death	
Cause of mortality	
